# Case Report: A novel KRT74 variant in an eight-year-old boy with alopecia totalis successfully treated with baricitinib

**DOI:** 10.3389/fmed.2025.1574656

**Published:** 2025-07-22

**Authors:** Yidong Tan, Jinxiang Yang, Xuanyi Chen, Yihang Shen, Zhe Wu, Weinan Zhou, Zhirong Yao, Jingjun Zhao, Jianying Liang

**Affiliations:** ^1^Department of Dermatology, Shanghai Jiao Tong University School of Medicine, Shanghai, China; ^2^Institute of Dermatology, Shanghai Jiaotong University School of Medicine, Shanghai, China

**Keywords:** alopecia totalis, KRT74 variant, JAK1-STAT1 pathway, leukonychia, baricitinib

## Abstract

Alopecia areata is an autoimmune condition characterized by non-scarring hair loss, with genetic factors playing a significant role in disease susceptibility. We report the case of an 8-year-old boy with alopecia totalis harboring a heterozygous KRT74 variant. While his mother and brother share this variant, they do not exhibit alopecia. Immunofluorescence analysis revealed increased phosphorylation of the JAK1-STAT1 pathway and elevated T cell infiltration, predominantly CD4^+^ T helper cells, in the proband’s scalp. The patient responded favorably to baricitinib treatment (2 mg/day), showing significant hair regrowth within 1 month and continued improvement over 8 months. This case suggests that KRT74 variants may contribute to immune dysregulation in alopecia areata, highlighting the potential role of JAK inhibitors in genetically predisposed alopecia cases.

## Introduction

Alopecia areata is an autoimmune condition leading to non-scaring hair loss (1), with an estimated lifetime prevalence of approximately 2% (2). Clinically, alopecia areata presents in various forms, ranging from well-defined patches (patchy alopecia areata) to complete hair loss on the scalp (alopecia totalis), or loss of all body hair (alopecia universalis). Genetic factors significantly contribute to alopecia areata susceptibility (3, 4). In children, the prevalence of child patients with a family history is estimated to be between 10% and 51.6% (3, 5). Large-scale genetic studies have identified several genes associated with alopecia area, including those encoding keratins.

Keratins are the most abundant structural protein in keratinocytes and contribute to form keratin intermediate filaments (KIFs) through heterodimerization between type I (acidic) and type II (basic to neutral) keratins (6). Beyond maintaining skin integrity, keratins are involved in regulating immune responses and hair growth (7). Pathological variant in keratin genes, such as *KRT71* (6) and *KRT74* (8), have been linked to hereditary hair disorders. *KRT74* is expressed in the inner root sheath of human hair follicle and heterozygous variants in *KRT74* cause the autosomal-dominant wooly hair (ADWH), affecting hair texture (8). We present a case of an 8-year-old boy with a heterozygous *KRT74* variant who developed alopecia totalis. The patient responded favorably to baricitinib treatment, marking the first reported instance of a *KRT74*-mutated patient with hair loss treated with baricitinib.

## Case presentation

**FIGURE 1:**
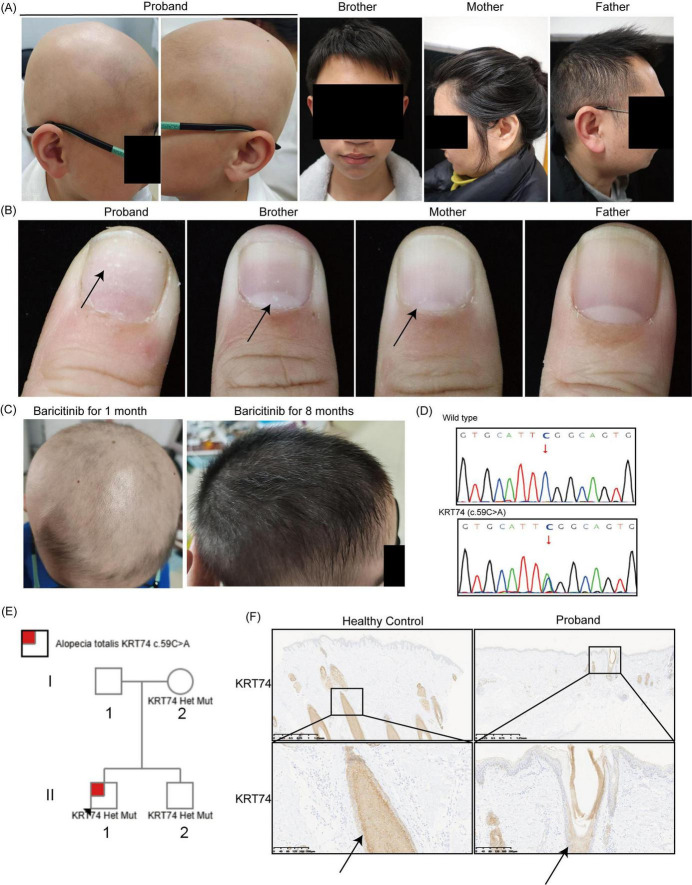
Clinical, genetic, and histopathological features of the proband and family members. **(A)** Clinical photographs of the proband, his brother, mother, and father. The proband exhibits alopecia totalis, while the other family members show normal hair growth. **(B)** Leukonychia (white spots on nails) in the nail of the proband, his brother, and mother, but not in the father. Arrows indicate the leukonychia. **(C)** Scalp images of the proband after treatment with baricitinib (2 mg/day), showing significant hair regrowth after 1 month and continued improvement after 8 months. **(D)** Sanger sequencing results identifying a heterozygous c.59C > A variant in the Keratin 74 (KRT74) gene. **(E)** Pedigree analysis of the family. **(F)** Immunohistochemical staining of scalp tissue showing reduced KRT74 expression in the proband’s hair follicles compared to a healthy control. Arrows indicate the expression of KRT74 in the hair follicle. Magnifications: 2× and 10×.

An 8-year-old boy presented with a 7-month history of alopecia totalis. Hair loss began 1 year prior, progressing to complete scalp hair loss within 5 months (Figure 1A). Initial treatments with vitamin B6 and glutamic acid supplements were ineffective in local hospital. Given the strong association between alopecia and genetic variants, particularly in pediatric cases (3), whole exome sequencing (WES) was performed, revealing a heterozygous *KRT74* variant (c.59C > A) (Figure 1D). Immunohistochemical analysis demonstrated downregulated expression of KRT74 in the patient’s hair follicle compared to a healthy control (Figure 1F). Pedigree analysis identified the same *KRT74* variant in the proband’s mother and brother (Figure 1E); however, neither exhibited alopecia (Figure 1A). The proband had normal eyebrows, eyelashes, sweating, and teeth with no signs of palmoplantar hyperkeratosis or keratosis pilaris. Notably, the patient exhibited leukonychia (white spots on nails), a feature also observed in his mother and brother, which has not been previously associated with *KRT74* variants (8, 9) (Figure 1B).

**FIGURE 2:**
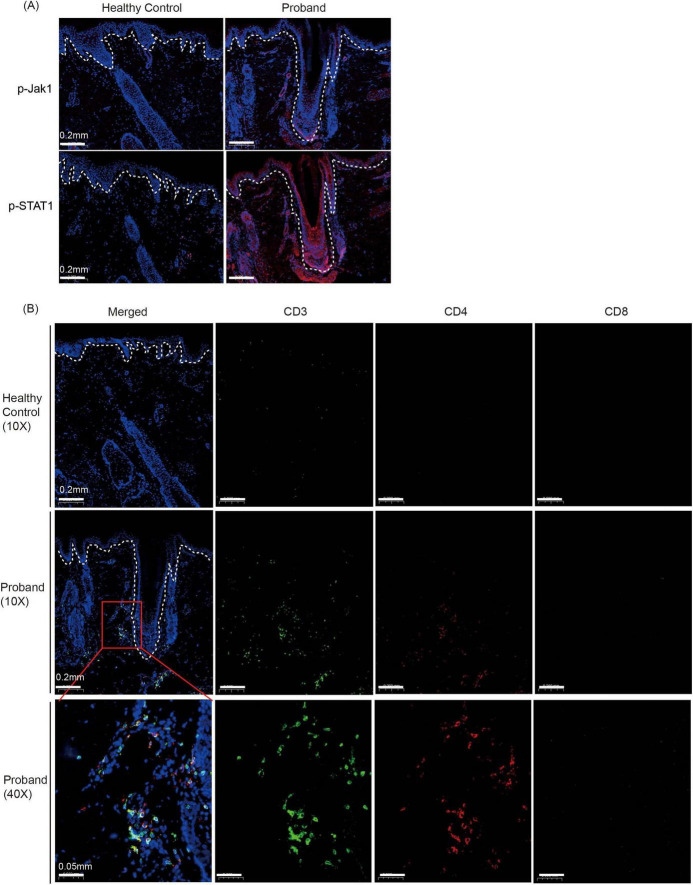
Immunofluorescence analysis of JAK-STAT pathway activation and immune cell infiltration in the proband’s scalp tissue. **(A)** Immunofluorescence staining for phosphorylated JAK1 (p-JAK1) and phosphorylated STAT1 (p-STAT1) in scalp tissue from a healthy control and the proband. Dashed lines demarcate the epidermal-dermal junction. Magnifications: 10×. **(B)** Immunofluorescence staining for immune cell markers in scalp tissue from a healthy control and the proband. Sections were stained for CD3 (green, T cells), CD4 (red, T helper cells), and CD8 (pink, cytotoxic T cells). The merged images show increased T cell infiltration in the proband’s scalp, with a predominance of CD4 + T helper cells. Dashed lines demarcate the epidermal-dermal junction. Magnifications: 10× and 40×.

Recent researches have demonstrated that alopecia is a disease that happens when the immune system attacks hair follicle and cause hair loss (10). Immunosuppressive treatments, such as baricitinib (11), dupilumab (12) and ritlecitinib (13) have proven effective. Variants in keratin genes can affect the immune homeostasis of keratinocyte (7). We hypothesized that the KRT74 variant might similarly affect the patient’s immune response. Using immunofluorescence, we analyzed the phosphorylation of Janus kinase (JAK) and STAT signaling pathway. The JAK1-STAT1 signaling is known to be activated in alopecia, and targeting the pathway has proved to be effective (14). We found the increased phosphorylation of JAK1 and STAT1 in the proband (Figure 2A). T cells are regarded as the primary effector cells in the pathogenesis of alopecia, particularly CD8^+^ cytotoxic T lymphocytes (15). To assess immune infiltration in the proband, we performed immunofluorescence staining for CD3, CD4, and CD8 markers. Our analysis revealed an increased presence of T cells (CD3^+^) in the proband. Interestingly, T helper cells (CD3^+^ CD4^+^) constituted the majority of T cell population, while the frequency of CD8^+^ cytotoxic T cell was not elevated compared with healthy controls (Figure 2B).

Given the increased JAK1 phosphorylation and the established efficacy of baricitinib in treating alopecia areata, the patient was initiated on baricitinib (2 mg/day). Significant hair regrowth was observed within 1 month (Figure 1C). Over an 8-months follow-up, the patient’s hair continued to thicken, indicating sustained treatment efficacy.

## Discussion

Keratin 74 is a type II keratin protein predominantly expressed in the inner root sheath of hair follicles, playing a crucial role in hair structure and integrity (9). Previous research has demonstrated that variants in *KRT74* are associated with autosomal dominant wooly hair and hypotrichosis (8). In our case study, we identified a heterozygous variant in *KRT74* in an 8-years-old patient presenting with alopecia totalis, an immune-mediated disorder. The variant c.59C > A results in a nonsense mutation at codon 20 (p.Ser20*), introducing a premature stop codon in exon 1 of KRT74. To assess the potential pathogenicity of this variant, using MutationTaster,^1^ the variant was classified as “Disease Causing” with a prediction score of 6.0, strongly suggesting its pathogenicity. The absence of alopecia in the proband’s mother and brother, who share the same *KRT74* variant, suggests potential incomplete penetrance or polygenic modulation of the phenotype. Our finding is particularly noteworthy as we observed activation of the JAK1-STAT1 pathway and T cell infiltration in the scalp of the patient with the *KRT74* variant. This suggests that KRT74 may influence immune response of the scalp.

The exact mechanism by which *KRT74* variants contribute to alopecia areata pathogenesis remains to be elucidated. It is plausible that alterations in keratin structure could disrupt the hair follicle’s immune privilege, rendering it more susceptible to autoimmune attacks. This concept is supported by studies on other keratin proteins; for instance, loss-of-function mutations in *KRT32* lead to hyperactivation of NF-κB signaling and are implicated in the pathogenesis of pityriasis rubra pilaris, an inflammatory skin disorder (7). Given the structural and functional similarities among keratin proteins, it is conceivable that *KRT74* variants may similarly influence immune regulatory pathways within hair follicles. Previous studies have suggested that self-reactive NKG2D^+^ CD8^+^ T cells and the activation of IFN-γ are key factors in the pathogenesis of alopecia areata (AA) (16, 17). However, recent studies have shown that CD4^+^ T cells may play a crucial role in the systemic Th1/Th2 inflammation and generalized phenotypes of AA patients (18–20). Our findings suggest that keratin mutations may preferentially activate CD4^+^ T cell-dependent pathways. We speculate that this could explain the clinical efficacy of JAK inhibitors (targeting signal transduction) observed in our cases. Further research is warranted to explore this potential connection and to determine whether *KRT74* variants could serve as biomarkers for alopecia areata susceptibility or targets for therapeutic intervention.

In addition to alopecia totalis, our patient exhibited leukonychia, characterized by white patches on the nails. KRT74 expression has been detected in the nail matrix, nail bed, and hyponychium, suggesting a role in nail development (21). The presence of leukonychia in our patient, along with similar findings in his mother and brother who harbor the same *KRT74* variant, suggests a potential association between this genetic alteration and nail pathology. This expands the phenotypic spectrum of *KRT74*-related disorders, which have primarily been associated with hair abnormalities. Further research is needed to elucidate the mechanisms by which *KRT74* variants contribute to nail manifestations and to determine the prevalence of such features among affected individuals.

Baricitinib, an oral JAK inhibitor, has emerged as a promising treatment for inflammatory conditions, including severe alopecia areata (11). Notably, its application has extended to genetic disease such as Aicardi-Goutières syndrome (22) and refractory inflammatory skin disease (23). Although in China, there is no specific regulation or guideline regarding the dosage of baricitinib for treating pediatric alopecia areata, these instances underscore baricitinib’s potential beyond its initial indications and its success in such contexts suggests a broader applicability for JAK inhibitors in managing genetically driven inflammatory conditions.

Our findings should be interpreted cautiously. First, the *KRT74* variant’s association with alopecia may reflect genetic susceptibility rather than direct causality, necessitating functional validation. Second, the lack of alopecia in family members underscores the potential role of gene-environment interactions, which were not systematically assessed here. Larger cohorts with longitudinal data are needed to clarify whether *KRT74* variants represent a risk modifier in polygenic alopecia areata.

In conclusion, our findings suggest that *KRT74* variants may play a role in the development of alopecia areata, highlighting the need for further studies to investigate this association. Elucidating the genetic underpinnings of alopecia areata can enhance our understanding of its pathophysiology and potentially lead to more effective, personalized therapeutic strategies.

## Data Availability

The original contributions presented in this study are included in this article/supplementary material, further inquiries can be directed to the corresponding authors.

## Footnotes

1
https://www.mutationtaster.org/
